# Sander’s Mission

**Published:** 1883-10

**Authors:** 


SANDERS' MISSION.—We record with pleasure the return of Mr. J. H. Sanders, of Chicago, who, as our readers will remember, was sent to Europe on a special mission in the interest of live stock, and tender him our congratulations on the successful result of his labors.

